# Machine Learning-Based Systems for the Anticipation of Adverse Events After Pediatric Cardiac Surgery

**DOI:** 10.3389/fped.2022.930913

**Published:** 2022-06-27

**Authors:** Patricia Garcia-Canadilla, Alba Isabel-Roquero, Esther Aurensanz-Clemente, Arnau Valls-Esteve, Francesca Aina Miguel, Daniel Ormazabal, Floren Llanos, Joan Sanchez-de-Toledo

**Affiliations:** ^1^BCNatal—Barcelona Center for Maternal-Fetal and Neonatal Medicine, Hospital Sant Joan de Déu and Hospital Clínic, University of Barcelona, Barcelona, Spain; ^2^Cardiovascular Diseases and Child Development, Institut de Recerca Sant Joan de Déu, Esplugues de Llobregat, Spain; ^3^Department of Pediatric Cardiology, Hospital Sant Joan de Déu Barcelona, Esplugues de Llobregat, Spain; ^4^BCNMedTech, Universitat Pompeu Fabra, Barcelona, Spain; ^5^Innovation in Health Technologies, Institut de Recerca Sant Joan de Déu, Esplugues de Llobregat, Spain; ^6^Department of Engineering, Hospital Sant Joan de Déu Barcelona, Esplugues de Llobregat, Spain; ^7^Department of Informatics, Hospital Sant Joan de Déu Barcelona, Esplugues de Llobregat, Spain; ^8^Department of Critical Care Medicine, School of Medicine, University of Pittsburgh, Pittsburgh, PA, United States

**Keywords:** artificial intelligence, machine learning, pediatric cardiology, intensive cardiac care, congenital heart disease, early warning score (EWS), risk stratification

## Abstract

Pediatric congenital heart disease (CHD) patients are at higher risk of postoperative complications and clinical deterioration either due to their underlying pathology or due to the cardiac surgery, contributing significantly to mortality, morbidity, hospital and family costs, and poor quality of life. In current clinical practice, clinical deterioration is detected, in most of the cases, when it has already occurred. Several early warning scores (EWS) have been proposed to assess children at risk of clinical deterioration using vital signs and risk indicators, in order to intervene in a timely manner to reduce the impact of deterioration and risk of death among children. However, EWS are based on measurements performed at a single time point without incorporating trends nor providing information about patient's risk trajectory. Moreover, some of these measurements rely on subjective assessment making them susceptible to different interpretations. All these limitations could explain why the implementation of EWS in high-resource settings failed to show a significant decrease in hospital mortality. By means of machine learning (ML) based algorithms we could integrate heterogeneous and complex data to predict patient's risk of deterioration. In this perspective article, we provide a brief overview of the potential of ML technologies to improve the identification of pediatric CHD patients at high-risk for clinical deterioration after cardiac surgery, and present the CORTEX traffic light, a ML-based predictive system that Sant Joan de Déu Barcelona Children's Hospital is implementing, as an illustration of the application of an ML-based risk stratification system in a relevant hospital setting.

## Introduction

Congenital heart disease (CHD) is the most common birth defect, accounting for almost 1% of all births (1) and remains a major contributor to early childhood morbidity and mortality. Healthcare advances have significantly decreased CHD infant mortality, yielding over 90% survival into adulthood. However, this has increased in- and out-of-hospital morbidity, exponentially increasing healthcare costs and caregiver burden. More than 80% of CHD surgical patients have cardiovascular complications such as arrhythmias and strokes during adulthood (2, 3). These complications affect functional and cognitive status and overall quality of life.

Compared to others, patients with CHD require more home health services, medical equipment and medication, e.g., hospitalization of infants with CHD represents 23% of global hospital resources even though accounts for only 4% of hospitalization (4). In addition, pediatric CHD patients are at higher risk of postoperative complications such as clinical deterioration, cardiac arrests, etc. either due to their underlying pathology, the cardiac surgery or the prolonged Intensive Care Unit (ICU) stay, contributing significantly to mortality, hospital stay, cost, and overall quality of life (5). In current clinical practice, anticipation of clinical deterioration in hospitalized pediatric patients remains challenging and, in most of the cases, adverse events are diagnosed when they have already occurred. Several early warning scores (EWS) exist for children. Common examples include the Pediatric Early Warning System (PEWS) score (6), the Brighton PEWS (7), and the Bedside PEWS (8), a simplified version of the PEWS. The Cardiac Children's Hospital Early Waring Score (C-CHWES), a modification of PEWS proposed for children with heart disease (9), has proven to be more sensitive and specific for identification of cardiac arrest and unplanned ICU transfer compared to PEWS (10). There are additional scores that have also been developed to predict mortality for intensive care patients, such as the PRISM-IV (11) and the PICSIM score (12). However, some of these EWS rely on subjective assessment making them susceptible to different interpretations, and have been unable to demonstrate a significant decrease in hospital mortality (13). Therefore, there is a need for the development of automated predictive models capable of capturing patient-specific data and predicting the risk of clinical decompensation continuously in real-time.

With the development of electronic health record (EHR) systems, large amounts of complex, high dimensional and heterogeneous data captured daily, are readily available for the development and validation of automated predictive models. By means of machine learning (ML)-based algorithms we could integrate data from different sources to predict which patients are at risk for clinical decompensation. In this article, we provide a brief overview of the potential of ML technologies for risk prediction in hospitalized children with a particular focus on CHD, since this population is at higher risk for clinical decompensation, cardiac arrest and mortality. Moreover, we present the CORTEX traffic light, an ML-based predictive system that Sant Joan de Déu (SJD) Barcelona Children's Hospital is implementing, as an illustration of the application of an ML-based system to stratify the risk of deterioration of pediatric CHD patients after cardiac surgery in a relevant hospital setting.

## Machine Learning to Predict Clinical Deterioration and/or Icu (Re-)Admission

Most adverse events in pediatric patients after cardiac surgery are preventable by early recognition of deterioration. For that reason, a number of different ML models have been proposed during the last 5–10 years to identify pediatric patients at risk of early postoperative clinical deterioration. We have summarized the publications on the use of ML in the prediction of clinical deterioration and unplanned ICU readmission in Supplementary Table 1.

Most of the postoperative adverse events are preceded by changes in patients' vital signs. Therefore, vital signs, such as heart rate (HR), respiration rate (RR), body temperature (BT), systolic (SBP), diastolic (DBP) and mean blood pressure (MBP) and oxygen saturation (SpO_2_) are the most common patient's variables used by the different authors to build their predictive models (13–25), with different levels of resolution ranging from few seconds (18, 21) to few hours (13). Zhai et al. (25) were the first group proposing to use an ML model to predict ward-to-ICU transfer using automatically extracted EHR data. They implemented a logistic regression model with 155 variables extracted from 36 measurements including vital signs, to predict ICU transfer for children in acute wards within the first 24 h of hospital admission, which showed an 0.91 area under the receiver operating characteristic (AUROC) in the test set. Then, several other ML-based models were proposed to predict clinical deterioration and/or an unplanned ward-to-ICU transfer (13, 14, 17, 20, 22, 26), adverse events within the ICU (15, 18, 19, 21, 23, 24, 27) or need for critical care within the emergency department (28). However, the best results were achieved when vital signs data were combined with other variables such as medications and/or laboratory test data as shown by Ruiz et al. (15). They developed an ensemble of 5 extreme gradient boosting models, using a total of 1,028 regularly collected EHR variables (vital signs, medications, laboratory tests and diagnosis) to identify patients in the cardiac ICU at elevated risk of clinical deterioration. Although they validated the model in a small cohort, the resulting model achieved an AUROC of 0.92 at 4 h before deterioration (15).

Regarding ML models specifically developed for children with CHD, most of the research has been focused on neonates with single ventricle, since these patients remain at significant risk of clinical deterioration and death compared to other CHD. Rusin et al. (24) implemented a multivariate logistic regression model to predict cardiorespiratory deterioration using six vital signs from 25 children with parallel circulation admitted to the ICU between early neonatal palliation and stage 2 surgical palliation. The proposed model showed the best performance approximately 1 to 3 h before deterioration (AUROC = 0.91). However, the study cohort was quite small, and all the subjects were from a single center. Ruiz et al. developed first a set of naïve Bayesian models to predict critical events in infants with single-ventricle physiology. The model was developed using 34 variables, including vital signs and laboratory data, from 93 children admitted to the ICU, and evaluated at 5 different time points before the onset of critical events. Their model was able to detect critical events 1 h in advance with AUROC of 0.88. However, all the subjects were from a single institution and therefore, their results may not be generalizable to different populations or institutions. More recently, the same authors presented an improved version of their model, developed using a bigger cohort of 488 patients with single-ventricle physiology, showing also very good results as discussed above (15).

On the other hand, Gu et al. (29) developed a multivariate logistic model for the prediction of postoperative risk in children with coarctation of the aorta (CoA), using data from 514 patients from two centers. However, they only used nine clinical, patient demographic, and surgical related variables such as incision of left thoracotomy, preoperative ventilation, etc. without including vital signs monitoring data. Their final model achieved an AUROC of 0.82 (29).

## Machine Learning to Predict Mortality

Several ML-based models have been already developed for mortality prediction in ICU, especially for adult population (30–34), based on large public available ICU datasets such as Medical Information Mart for Intensive Care (MIMIC-III) (35) and eICU Collaborative Research Database (eICU) (36). However, few ML-based models have been specifically developed for pediatric ICU. We have summarized the studies conducted to predict mortality in the pediatric population in Supplementary Table 1.

Most of the proposed models were developed to make one prediction per patient encounter using data within the first hours after ICU admission (37–40), rather than predict the risk of mortality continuously across the entire encounter. However, in order to continually assess individual patient's risk of clinical deterioration or mortality it is important to integrate information not only from a single time point, as the current scoring systems do, but also data from previous time points, that is, longitudinal temporal data. For example, it is more critical and useful for the clinician to know the speed of the decrease in oxygen saturation level, or how much time oxygen saturation level has been below patient's baseline for the last hour rather than knowing that oxygen level is low at a specific time point. For that reason, ML models based on recurrent neural networks (RNN), such as long short-term memory (LSTM) models have been proposed to continuously assess the individual child's risk of mortality thought their hospital admission. This type of ML models can process entire sequence of data, thus allowing retention of information from previous times and integration with newly acquired data to make a new prediction. Aczon et al. (41, 42) developed a LSTM model using 430 distinct physiologic, demographic, laboratory, and therapeutic variables, to provide individual patient's mortality risk at any time during their ICU admission when a recorded measurement became available. The authors evaluated the performance of their model at various time points from 0 to 24 h after ICU admission, as well as, from 1 to 24 h prior to discharge. Their model performed well showing an AUROC of 0.99 24 h prior to discharge. However, they only used data from a single center to develop and validate their model, thus limiting its generalizability to different institutions. Ho et al. (43) partially investigate the problem of ML models generalization and their dependence on the data quality, by emulating different permutations of EHR data collected from two different ICUs from the same children's hospital. They showed a large performance disparity between the two different test sets across all data permutations and algorithms. Among the different ML models evaluated, their results indicated that the multilayer perceptron model learned more generalizable patterns than the RNN, maybe due to the larger number of parameters used by the RNN, which could lead to overfitting of the data (43).

Other authors proposed to use perioperative data to predict the risk of mortality after cardiac surgery in pediatric patients with CHD. For example, Bertsimas et al. developed ML models to predict mortality, need for postoperative mechanical ventilatory support and prolonged length of stay (LOS) for patients with CHD that underwent cardiac surgery. The model was based on preoperative data from more than 235,000 patients, but without including vital signs data (44). Their model based on optimal classification trees achieved a mortality AUROC of 0.86. Jalali et al. (45) used a Chain Monte-Carlo simulation method to impute missing data and developed and tested five ML models to predict the individualized risk of prolonged LOS and risk of mortality or cardiac transplantation at 1-year after the Norwood surgical procedure. They used preoperative and intraoperative data from 549 newborns with single ventricle physiology to train the five ML models. The best results were obtained with the deep neural network (DNN) model that demonstrates an AUROC of 0.95 for 1-year mortality or cardiac transplantation and 0.94 for prolonged LOS.

Although deep learning-based models outperformed more traditional ML-based models as illustrated in most of the publications discussed here, their applicability in a real clinical setting is still very scarce mainly because they are considered “black-box” models due to their complexity and lack of interpretability with the inherent difficulty of providing an intuitive clinical explanation of the proposed prediction. Therefore, other explainable ML approaches able to integrate complex and heterogeneous time-varying data, based on for example the identification of individuals with similar patterns, seem more promising (46–49).

## The Cortex “Traffic Light”

CHD affects approximately 108,000 new-borns each year in EU and over 2 M EU citizens. Approximately 25% of them will require surgical or trans-catheter intervention during their first year of life (1, 50). Unfortunately, about half of these patients suffer from neuro-developmental impairments that expand through adulthood. To address this, in 2020 SJD Barcelona Children's Hospital launched CORTEX “traffic light,” a CHD patient stratification score system fed with EHR patient data used in daily practice for CHD management in SJD. This scoring system automatically extracts real-time data from EHR (e.g., HR, SpO_2_, cardiac physiology, etc.) to stratify patient risk of clinical deterioration during the entire patient journey after cardiac surgery (ICU, ward and home). The risk is then rated *via* a traffic light scorecard (green = stable; yellow = at risk; red = unstable), allowing optimal decision-making (e.g., early discharge from ICU if green light after surgery). The scorecard appears on the dashboard of the SJD command center. The first and current version of CORTEX “traffic light” consisted of a set of rules applied to six vital sign monitoring variables (HR, SpO_2_, RR, SBP, DBP, and BT) as a function of patient's age and cardiac physiology (cyanotic/non-cyanotic CHD) (see Supplementary Table 2). More details about CORTEX “traffic light” are provided in Supplementary Methods.

A 1-year pilot study was conducted in SJD (from September 2020 to September 2021) to monitor CHD pediatric patients at the ward using CORTEX “traffic light”, after surgical or transcatheter intervention. A total of 254 pediatric patients with a total of 294 episodes were monitored during the pilot study. Table 1 shows the clinical and demographic data, including the average LOS and number of adverse events (AE) experienced by the patients during their ward admission. Of the 254 subjects, 41 (16.14%) experienced one or more AE on the ward but only 8 (3.15%) were followed by an unplanned ward-to-ICU transfer (Table 1). Twenty-five (47.2%) were primarily cardiac events in nature, whereas 12 (22.6%) were considered respiratory events. There was one hospital death in the cohort. As seen in Table 1, those patients experiencing an AE at ward have significant longer hospital LOS. The description of the statistical analysis can be found in the Supplementary Methods.

**Table 1:** Demographic and clinical data of the patients included the 1st year pilot study of CORTEX “traffic light”.

**Variables**	**Control group**	**AE group**	***p*** -value^*^	**AE without ICU**	***p-*** value^*^	**AE with ICU**	***p-*** value^*^
	**(*N* = 253)**	**(*N* = 41)**		**transfer** **(*N* = 33)**		**transfer (*N* = 8)**	
Age	7.02 ± 6.08	5.08 ± 5.46	0.056	5.82 ± 5.51	0.083	2.02 ± 4.31	**0.006**
Sex, female *n* (%)	107 (42.3%)	11 (26.8%)	0.061	9 (27.3%)	0.098	2 (25%)	0.329
LOS (days)	1.6 [1.0, 7.0]	9.9 [7.5, 25.1]	**<0.001**	9.2 [6.9, 13.7]	**<0.001**	43.3 [15.9, 62.9]	**<0.001**
Cyanotic CHD *n* (%)	56 (22.1%)	15 (36.6%)	**0.045**	10 (30.3%)	0.295	5 (62.5%)	**0.008**
**Number of adverse events**			**<0.001**		**<0.001**		**<0.001**
0	253 (100%)	0 (0%)		0 (0%)		0 (0%)	
1	0 (0%)	34 (83%)		2 (84.8%)		6 (75%)	
2	0	5 (12%)		4 (12.1%)		1 (12.5%)	
2 +	0	2 (5%)		1 (3.0%)		1 (12.5%)	
Death	0 (0%)	1 (2%)	–	0 (0%)	–	1 (12.5%)	–

*AE, adverse event; LOS, Length of stay; CHD, Congenital Heart Disease; Cyanotic CHD includes the following defects, common arterial trunk (before corrective surgery), double outlet right ventricle (before corrective surgery), double outlet left ventricle, transposition of the great arteries, double inlet ventricle, pulmonary valve atresia, pulmonary valve stenosis, tricuspid valve stenosis, Ebstein's anomaly, hypoplastic right heart syndrome, hypoplastic left heart syndrome, pulmonary infundibular stenosis, subaortic stenosis, atresia of aorta, interruption of the aortic arch, atresia of pulmonary artery, stenosis of pulmonary artery, congenital pulmonary arteriovenous malformation and total anomalous pulmonary venous connection. Continuous variables are expressed as mean ± standard deviation or median [25th−75th percentile] based on a normal distribution by Kolmogorov-Smirnov testing. Categorical variables are presented as n (%)*.

* *As compared to control group. Bold values denote statistical significance at the p < 0.05 level*.

*AE group accounts for all the patient encounters in which CHD patients experienced one or more adverse events (AE). We then split AE group into two subgroups: AE without ICU transfer group, which includes all the patient encounters in which a patient experiences an AE without an unplanned ward-to-ICU transfer; and AE with ICU transfer, which includes all the patient encounters in which a patient experiences an AE followed by an unplanned ward-to-ICU transfer*.

In order to retrospectively evaluate the performance of CORTEX “traffic light” within 1st year pilot study, we compared the score between control group, defined as ward admissions during which patients did not experience AE, and experimental group, defined as ward admissions during which patients experienced one or more AE. We further divided the experimental group into two subgroups: those patients who experienced one or more AE followed by an unplanned ward-to-ICU transfer and patients who experienced one or more AE without an unplanned ward-to-ICU transfer. Individual episodes for patients with multiple admissions were considered independently. We computed the average CORTEX “traffic light” score for each patient admission in a 30-min window, from 1 to 8 h before the AE, in 1 h intervals. Because controls did not have an associated AE time, a time point was selected randomly within their whole ward admission excluding the first 8 h after admission.

**Figure 1:**
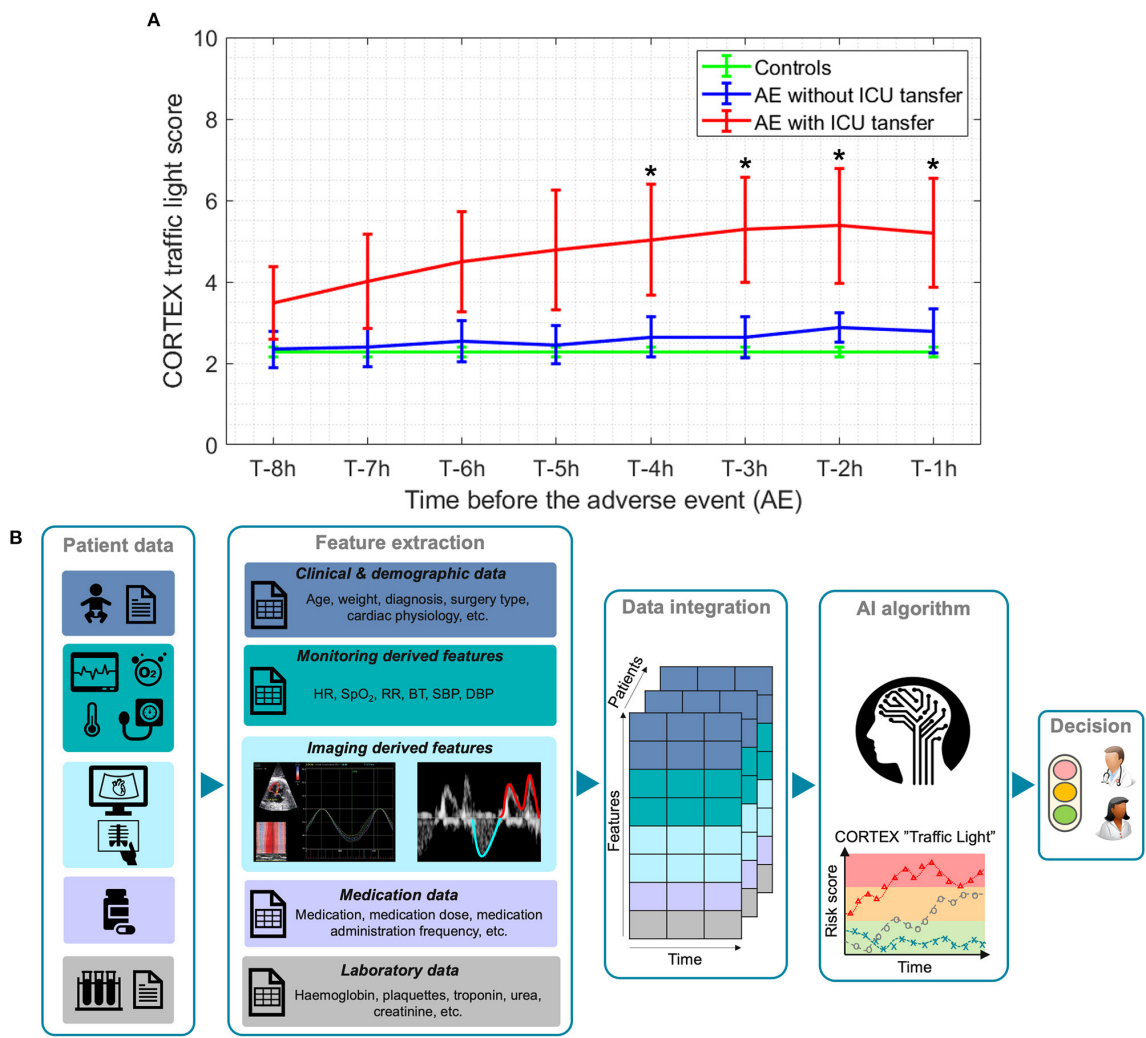
**(A)** Comparison of average CORTEX “traffic light” score, from 1 to 8 h before the adverse event (AE) between controls (green), patients who experience one or more AEs without an unplanned ward-to-ICU transfer (blue) and patients who experience one or more AEs followed by an unplanned ward-to-ICU transfer (red). T denotes the time when an AE occurs. Lines: group means; whiskers: +/- standard error. ^*^ Significantly different from the control group, *p* < 0.05. **(B)** The overall pipeline of CORTEX “traffic light” machine learning-based algorithm for the risk stratification of CHD pediatric patients.

Figure 1A shows the average CORTEX “traffic light,” from 1 to 8 h before the AE, calculated in 1 h intervals, for the three study groups [controls: patients who did not experience any AE (green), patients who experienced an AE but without an unplanned ward-to-ICU transfer (blue) and patients who experienced an AE followed by an unplanned ward-to-ICU transfer (red)]. As illustrated in this graph, the average, CORTEX “traffic light” score of those children who experienced an unplanned ward-to-ICU transfer was significantly higher compared to controls already 4 h before the AE (2.27 vs. 5.03, *p* = 0.037). Moreover, we evaluated whether CORTEX “traffic light” has helped us to optimize patient allocation of resources during this 1st year pilot study. To do that, we compared ICU and hospital LOS of CHD patients admitted to our hospital within the same period before the implantation of SJD CORTEX and before the COVID19 pandemic. A reduction of CHD ICU and hospital LOS by 7 and 8% respectively with associated hospital savings of 138 k was observed.

We are currently working on the implementation of an improved version of CORTEX “traffic light” by integrating more than 50 patient's variables including data not only from monitoring but also from laboratory, medications, echocardiography, etc. by means of interpretable ML algorithms based on representation learning (through non-linear dimensionality reduction and manifold learning) (47–49, 51), as illustrated in Figure 1B. Once fully validated, CORTEX “traffic light” will be used to continuously monitor CHD pediatric patients, and predict their individual risk of clinical deterioration throughout hospital admission and even at home. This will also help to allocate appropriate care treatment, use resources more efficiently and improve patient and family satisfaction, with an expected impact on clinical outcomes and healthcare costs.

## Conclusions

Pediatric patients with CHD are at higher risk for clinical deterioration, and early recognition of this can improve outcomes and prevent mortality. ML models that aggregate multimodal patient data to discriminate different patterns and identify subacute stages of clinical decompensation, and taking into account patient longitudinal data, are promising for individual risk stratification and outcome prediction in children with CHD after cardiac surgery.

We believe that, in the near future, ML algorithms will become integrated into our hospitals, thus improving patient management, allocation of healthcare resources and ultimately individualized clinical care. However, lack of interpretability in predictive models, such as the commonly referred to as “black-box models” can undermine trust in ML models, especially in healthcare. Therefore, care must be taken to ensure that models are not only accurate but can also provide interpretable explanations to the clinician as to why they have given a particular result, to facilitate the understanding of the model's predictions.

In the setting of CHD, with very heterogeneous presentations, interpretable ML models based on comparing the current patient with previously known ones or with themselves during their temporal trajectory, by presenting a computed information similarity, can help clinicians in their decision-making, for prognosis and therapy prediction.

## Data Availability Statement

The original contributions presented in the study are included in the article/Supplementary Material, further inquiries can be directed to the corresponding author.

## Ethics Statement

The studies involving human participants were reviewed and approved by the Local Institution's Ethics Committee (PIC-249-20). Written informed consent from the participants' legal guardian/next of kin was waived by the local Ethical Committee.

## Author Contributions

All authors listed have made a substantial, direct, and intellectual contribution to the work and approved it for publication.

## Funding

PG-C has received funding from the postdoctoral fellowships program Beatriu de Pinos (2018-BP-00201), funded by the Secretary of Universities and Research (Goverment of Catalonia) and by the Horizon 2020 programme of research and innovation of the European Union under the Marie Skłodowska-Curie Grant Agreement No. 801370. AI-R has received funding from the Pla de Doctorats Industrials de la Secretaria d'Universitats i Recerca del Departament d'Empresa i Coneixement de la Generalitat de Catalunya (2021 DI 88). This Project has received funding from the European Union's Horizon 2020 research and innovation programme under Grant Agreement No. 101016902.

## Conflict of Interest

The authors declare that the research was conducted in the absence of any commercial or financial relationships that could be construed as a potential conflict of interest.

## Publisher's Note

All claims expressed in this article are solely those of the authors and do not necessarily represent those of their affiliated organizations, or those of the publisher, the editors and the reviewers. Any product that may be evaluated in this article, or claim that may be made by its manufacturer, is not guaranteed or endorsed by the publisher.
