# Supplementary material for: Machine Learning-Based Systems for the Anticipation of Adverse Events After Pediatric Cardiac Surgery
Source: Front Pediatr. 2022 Jun 27;10:930913. doi: 10.3389/fped.2022.930913 (PMC9271800; doi:10.3389/fped.2022.930913)
Supplement: Supplementary file 1 [file Data_Sheet_1.docx]

Supplementary Material

# Supplementary Methods

## CORTEX “traffic light”

Sant Joan de Déu (SJD) Barcelona Children’s Hospital CORTEX eCare is a centralized advanced cardiac monitoring center for pediatric patients, launched in 2020, which aims to have a remote readily available continuous monitoring system of patient’s health during the whole recovery period after a cardiac procedure. CORTEX eCare is operated by a multidisciplinary group of professionals incusing clinicians, data scientists, biomedical engineers and informaticians that work together to build a risk stratification score system fed with electronic health record (EHR) patient data, CORTEX “traffic light”, for the management of patients with congenital heart disease (CHD). The first version of CORTEX “traffic light” score system is based on ruled-based model applied to the following six vital signs: heart rate (HR), oxygen saturation (SpO_2_), body temperature (BT), respiration rate (RR), systolic blood pressure (SBP) and diastolic blood pressure (DBP), as a function of age and cardiac physiology of the patient. CORTEX “traffic light” is already being used to monitor CHD patients at the ward in SJD. A telemetry wireless monitor (BeneVision TM80 from Mindray) is used to automatically measure ECG-derived HR, RR and SpO_2_ every minute during patient’s ward admission, while SBP, DBP and BT are measured by the nurses every 8h and introduced manually into the EHR system. All the six variables are automatically collected and stored in a dedicated database (Digistat Smart Central from ASCOM). For each vital sign variable, and depending on the age and cardiac physiology of the patient (cyanotic or non-cyanotic CHD), a score, ranging from 0 to 3, is calculated as described in **Supplementary Table 2**. Then, the final score is calculated as the addition of all six individual scores. Every time a new patient’s measurement becomes available, CORTEX “traffic light” score is calculated. Finally, three different colors are used to represent patient's last CORTEX “traffic light” score on CORTEX eCare board as follows: green: 0-1, yellow: 2-3 and red: >3, providing easy visibility and understating of all the patients' CORTEX “traffic light” score for clinicians.

## Statistical analysis

Continuous variables were expressed as mean±standard deviation or median [25th–75th percentile] based on a normal distribution by Kolmogorov-Smirnov testing. Categorical variables were presented as n (%). Differences between groups (controls vs experimental groups) were analysed for statistical significance using bilateral independent t-test for normally distributed variables, Kruskal-Wallis for non-normally distributed variables, and Chi-square test for categorical variables. Analyses were performed using SPSS (version 28.0 for Windows) and p-value<0.05 indicated statistical significance.

# Supplementary Tables

**Supplementary Table 1**. Summary of all the publications about the use of ML-based models to predict the risk of clinical deterioration and mortality in hospitalized children.

| **Author** | **Year** | **Nº of samples** | **Outcome** | **Variables** | **Setting** | **Methodology** | **Bets results (AUROC)** |
| --- | --- | --- | --- | --- | --- | --- | --- |
| ***Clinical deterioration and unplanned ward-ICU transfer*** | | | | | | | |
| Park SJ et al. (1) | 2021 | 50,019 (813 with outcome) | Cardiopulmonary arrest (CPA) & unplanned ward-to-ICU transfer | Demographics, clinical data and 5 vital signs (HR, RR, SBP, DBP, BT) | Ward | Bidirectional LSTM | 0.92 (CPA); 0.91 (unplanned ICU transfer) |
| Comoretto RI et al.(2) | 2021 | 29,494 (399 with outcome) | Hemodynamic failure | Clinical characteristics at admission | ICU | GLM, RPART, RF, NN, XGBoost | 0.78 (XGboost) |
| Ruiz VM et al. (3) | 2021 | 488 (203 with outcome) | Clinical deterioration | 1028 variables including vital signs, medications, laboratory test and diagnoses. | ICU | Ensemble of 5 XGBoost models | 0.92 (4h before deterioration) |
| Gu Y et al. (4) | 2021 | 514 patients with CoA (195 with outcome) | Postoperative adverse events (death and complications) | 64 variables (demographics, clinical, imaging, surgical and laboratory data) | N/A | Multivariate logistic regression | 0.82 |
| Zeng X et al. (5) | 2021 | 1,964 (584 with outcome) | Postoperative complications | Demographic, clinical (diagnoses & procedures) and intraoperative data including vital signs | N/A | XGBoost and SHAP | 0.84 (all complications);  0.95 (cardiac complications) |
| Mayampurath A et al. (6) | 2020 | 38,199 (1375 with outcome) | Unplanned ward-to-ICU transfer | 10 variables (4 demographic variables and 6 vital signs: BT, RR, HR, SpO_2_, SBP and DBP) | Ward | Discrete-time logistic regression | 0.77 (6h before the event) |
| Izquierdo L et al. (7) | 2020 | 90,678 records from 90 patients | Clinical deterioration | 6 vital signs (SBP, DBP, MB, HR, RR and SpO_2_) | ICU | Hidden Markov Model + k-means | Mean accuracy = 0.7* |
| Gorham TJ et al. (8) | 2020 | 135,755 encounters (158 with outcome) | Clinical deterioration (Unplanned ward-do-ICU transfer and “code blue” events) | 5 vital signs + supplemental oxygen requirement | Ward | Multivariate logistic regression | 0.76 vs 0.73 for PEWS |
| Zeng X et al. (9) | 2019 | 2,308 patients (677 with outcome) | Postoperative complications | Demographic, preoperative clinical, surgery and postoperative data | N/A | XGBoost | 0.82 vs 0.75 for STS-EACTS score. |
| Ruiz VM et al. (10) | 2019 | 93 patients with single-ventricle physiology with 146 encounters (131 with outcome) | Postoperative adverse events | 34 variables including clinical, vital signs and laboratory data | ICU | Naïve Bayes network models | 0.88 (1h before the event) |
| Berry JG et al. (11) | 2019 | 3,295 patients (882 with outcome) | Clinical deterioration | Demographics and number of complex chronic conditions and chronic medication | Ward | Multivariate logistic regression | N/A (adjusted odds-ratio = 1.2) |
| Kwon JM et al. (12) | 2019 | 2,937,078 patients (18,255 with critical care and 375,079 with hospitalizations) | Need for critical care (primary) and hospitalization (secondary) | Demographics, clinical and initial vital signs (HR, RR, BT) data | ED | MLP | 0.91 for predicting critical care; 0.78 for predicting hospitalizations. |
| Rubin J et al. (13) | 2018 | 15,427 patients with 18,973 encounters (432 with outcome) from 2 databases | Unplanned ward-to-ICU transfer | Demographics and vital signs data (HR, SpO2, RR, BT, DBP, SBP, MBO, Pulse pressure, Shock index). | Ward | AdaBoost and gradient tree boosting | 0.84 vs. 0.75 for modified PEWS |
| Matam B et al. (14) | 2018 | 3,435 patients (175 with outcome) | Cardiac arrest | 4 vital signs (HR, RR, SBP and SpO_2_) | ICU | Singular spectrum analysis + auto-regressive model | 0.75 |
| Wellner B et al. (15) | 2017 | >1 million patient with >8 million encounters (3,927 with outcome) from 3 hospitals | Unplanned ward-to-ICU transfer | 75 variables including vital sings, laboratory data, acuity scores and nurse assessments | Ward | Regularized logistic regression and MLP | 0.89, 0.91 and 0.89 for each institution with MLP (6h before the event) |
| Potes C et al. (16) | 2017 | 8,022 patients (3,180 with outcome) from 2 hospitals | Hemodynamic instability | 36 variables including vital signs, laboratory data and ventilator parameters | ICU | AdaBoost | 0.81 |
| Rusin C et al. (17) | 2016 | 25 patients with parallel circulation (13 with outcome) | Cardiorespiratory deterioration | 6 vital signs variables | ICU | Multivariate logistic regression | 0.91 (1-2h before the event) |
| Zhai H et al. (18) | 2014 | 71,752 patient encounters (526 with outcome) | Ward-to-ICU transfer within the first 24h | 155 variables including demographic, clinical and vital signs data | Ward | Logistic regression | 0.91 |
| ***Mortality*** | | | | | | | |
| Lee B et al. (19) | 2021 | 2,596 patients (78 with outcome) from 4 institutions | Mortality within 72h of admission | Demographic, ICU admission-related, clinical, and vital signs data | ICU | RF | 0.91 vs 0.85 for PIM3 (validation cohort) |
| Aczon M et al. (20) | 2021 | 9,070 patients with 12,516 encounters (472 with outcome) | Mortality | 430 variables including demographic, medications, laboratory, and vital signs data | ICU | LSTM | 0.94 12h after ICU admission; 0.99 24h before discharge/death |
| Hong S et al. (21) | 2021 | 13,258 patients (780 with outcome) | Mortality | 397 variables including demographic, input and output, laboratory vital signs data | ICU | Ensemble step-wise feature selection + logistic regression | 0.75 vs 0.69 PRISM 3 (using 11 features) |
| Prince R et al. (22) | 2021 | 9,980 patients with 14.237 encounters (443 with outcome) | Mortality | Variables used to calculi the PELOD-2 score such as Glasgow Coma Scale score, pupillary reaction, lactate, etc. and age | ICU | Decision trees, Naïve Bayes, SVM, KNN, boosted ensemble and RF | 0.87 for RF vs 0.76 for PELOD-2 |
| Bertsimas D et al. (23) | 2021 | >235,000 patients with +295,000 operations (2,950 with outcome) | Mortality, prolonged mechanical ventilatory support time (MVST) and prolonged hospital LOS. | Preoperative clinical variables | ICU | Optimal classification trees (OCT), logistic regression, RF and GBM | 0,86 (mortality), 0.85 (prolonged MVST) and 0.82 (prolonged LOS) with OCT. |
| Hu Y et al. (24) | 2021 | 1,481 patients (50 with outcome, 4.79% with CHD | Postoperative mortality | 261 variables including demographics, clinical data as well as vital signs during surgery (HR, SBP, DBP, MBP, BT and SpO_2_) | N/A | XGBoost, RF, logistic regression, Naïve Bayes and KNN + SHAP | 0.74 |
| Jalali A et al. (25) | 2020 | 549 patients with HLHS (72 with outcome) | 1-year mortality or cardiac transplantation and prolonged LOS | Preoperative and intraoperative variables | N/A | DNN, GBM, RF, DT and Ridge Regression | 0.95 (mortality/cardiac transplantation) and 0.94 (prolonged LOS) with DNN |
| Kim S et al. (26) | 2019 | 1,723 patients with 2,341 encounters (338 with outcome) | Mortality | Demographic, clinical and 7 vital signs data (SBP, DBP, MBP, HR, RR, SpO_2_ and BT | ICU | CNN | 0.96 6h prior to death |
| Miller R et al. (27) | 2018 | 3,180 (286 with outcome) | Mortality after heart transplant within 1 year | Demographic and clinical data from donor and recipient | N/A | Classification and regression trees, RF and NN | 0.74 with RF |
| Williams J et al. (28) | 2018 | 11,384 patient encounters (249 with outcome) | Mortality | 35 variables including demographic, clinical, laboratory, medications, and vital signs data | ICU | k-means | 0.77 |
| Ho L et al. (29) | 2017 | 17,268 patients with 21,881 encounters (389 with outcome) | Mortality within 12h | Demographic, clinical, laboratory, medications, and vital signs data | ICU | Logistic regression, MLP and LSTM | 0.92 for LSTM |
| Aczon M et al. (30) | 2017 | 12,020 patients with 16,559 encounters (444 with outcome) | Mortality | ~300 variables including demographic, clinical, laboratory, medications, and vital signs data | ICU | RNN | 0.93 |

AdaBoost: Adaptive Boosting; BT: body temperature; CNN: Convolutional neural network; CoA: Coarctation of the aorta; DBP: diastolic blood pressure; DNN: deep neural network; DT: decision tree; GBM: Gradient Boosting Machine; GLM: Generalized linear model; HLHS: Hypoplastic left heart syndrome; HR: heart rate; ICU: intensive care unit; KNN: K-Nearest Neighbors; LSTM: Long short-term memory; MBP: mean blood pressure; MLP: Multilayer perceptron; NN: neural network; RF: Random Forest; RNN: recurrent neural network; RPART: recursive partitioning; RR: respiration rate; SBP: systolic blood pressure; SHAP: SHapley Additive exPlanations; SpO_2_: oxygen saturation; SVM: Super vector machine; XGBoost: eXtreme Gradient Boosting.

**Supplementary Table 2. CORTEX “Traffic light” score**. For each vital sign variable and depending on the age and cardiac physiology of the patient (cyanotic or non-cyanotic congenital heart defect), a score, ranging from 0 to 3, is calculated. Then, the CORTEX “Traffic light” score is calculated as the addition of all six individual scores. A different traffic light colour is assigned as a function of total score as follows: green: 0-1, yellow: 2-3 and red: >3.

| **Variable** | **Non-cyanotic CHD** | | | | **Cyanotic CHD** | | | |
| --- | --- | --- | --- | --- | --- | --- | --- | --- |
|  | **0 points** | **1 point** | **2 points** | **3 points** | **0 points** | **1 point** | **2 points** | **3 points** |
| *Age group: 0 – 6 months* | | | | | | | | |
| HR (bpm) | ≥120 & <145 | ≥100 & <120 or  ≥145 & <160 | ≥95 & <100 or  ≥160 & <180 | <95 or ≥180 | ≥120 & <145 | ≥100 & <120 or  ≥145 & <160 | ≥95 & <100 or  ≥160 & <180 | <95 or ≥180 |
| RR (rpm) | ≥30 & <45 | ≥20 & <30 or  ≥45 & <55 | ≥55 & <65 | <20 or ≥65 | ≥30 & <45 | ≥20 & <30 or  ≥45 & <55 | ≥55 & <65 | <20 or ≥65 |
| Body Temp. (º) | ≥35,7 & <37 | ≥37,5 & <38,5 or  < 35,7 | ≥38,5 | - | ≥35,7 & <37 | ≥37,5 & <38,5 or  < 35,7 | ≥38,5 | - |
| SpO_2_ (%) | ≥94 & <100 | ≥90 & <94 | ≥85 & <90 | <85 | ≥80 & <90 | ≥75 & <80 or  ≥90 & <100 | ≥70 & <75 | <70 |
| SBP (mmHg) | ≥55 & <70 | ≥45 & <55 or  ≥70 & <80 | ≥35 & <45 or  ≥80 & <90 | <35 or ≥90 | ≥55 & <70 | ≥45 & <55 or  ≥70 & <80 | ≥35 & <45 or  ≥80 & <90 | <35 or ≥90 |
| DBP (mmHg) | ≥30 & <50 | ≥25 & <30 or  ≥50 & <60 | ≥20 & <25 or  ≥60 & <70 | <20 or ≥70 | ≥30 & <50 | ≥25 & <30 or  ≥50 & <60 | ≥20 & <25 or  ≥60 & <70 | <20 or ≥70 |
| *Age group: 6 – 12 months* | | | | | | | | |
| HR (bpm) | ≥120 & <165 | ≥100 & <120 or  ≥165 & <175 | ≥90 & <100 or  ≥175 & <185 | <90 or ≥185 | ≥120 & <165 | ≥100 & <120 or  ≥165 & <175 | ≥90 & <100 or  ≥175 & <185 | <90 or ≥185 |
| RR (rpm) | ≥20 & <35 | ≥35 & <45 | ≥45 & <55 | <20 or ≥55 | ≥20 & <35 | ≥35 & <45 | ≥45 & <55 | <20 or ≥55 |
| Body Temp. (º) | ≥35,7 & <37 | ≥37,5 & <38,5 or  < 35,7 | ≥38,5 | - | ≥35,7 & <37 | ≥37,5 & <38,5 or  < 35,7 | ≥38,5 | - |
| SpO_2_ (%) | ≥94 & <100 | ≥90 & <94 | ≥85 & <90 | <85 | ≥80 & <90 | ≥75 & <80 or  ≥90 & <100 | ≥70 & <75 | <70 |
| SBP (mmHg) | ≥70 & <90 | ≥60 & <70 or  ≥90 & <100 | ≥50 & <60 or  ≥100 & <110 | <50 or ≥110 | ≥70 & <90 | ≥60 & <70 or  ≥90 & <100 | ≥50 & <60 or  ≥100 & <110 | <50 or ≥110 |
| DBP (mmHg) | ≥45 & <65 | ≥35 & <45 or  ≥65 & <75 | ≥30 & <35 or  ≥75 & <80 | <30 or ≥80 | ≥45 & <65 | ≥35 & <45 or  ≥65 & <75 | ≥30 & <35 or  ≥75 & <80 | <30 or ≥80 |
| *Age group: 1 – 2 years* | | | | | | | | |
| HR (bpm) | ≥105 & <160 | ≥90 & <105 or  ≥160 & <175 | ≥175 & <195 | <90 or ≥195 | ≥105 & <160 | ≥90 & <105 or  ≥160 & <175 | ≥175 & <195 | <90 or ≥195 |
| RR (rpm) | ≥20 & <30 | ≥30 & <40 | ≥40 & <50 | <20 or ≥50 | ≥20 & <30 | ≥30 & <40 | ≥40 & <50 | <20 or ≥50 |
| Body Temp. (º) | ≥35,7 & <37 | ≥37,5 & <38,5 or  < 35,7 | ≥38,5 | - | ≥35,7 & <37 | ≥37,5 & <38,5 or  < 35,7 | ≥38,5 | - |
| SpO_2_ (%) | ≥94 & <100 | ≥90 & <94 | ≥85 & <90 | <85 | ≥80 & <90 | ≥75 & <80 or  ≥90 & <100 | ≥70 & <75 | <70 |
| SBP (mmHg) | ≥75 & <95 | ≥65 & <75 or  ≥95 & <105 | ≥55 & <65 or  ≥105 & <115 | <55 or ≥115 | ≥75 & <95 | ≥65 & <75 or  ≥95 & <105 | ≥55 & <65 or  ≥105 & <115 | <55 or ≥115 |
| DBP (mmHg) | ≥50 & <70 | ≥40 & <50 or  ≥70 & <80 | ≥35 & <40 or  ≥80 & <90 | <35 or ≥90 | ≥50 & <70 | ≥40 & <50 or  ≥70 & <80 | ≥35 & <40 or  ≥80 & <90 | <35 or ≥90 |
| *Age group: 2 – 6 years* | | | | | | | | |
| HR (bpm) | ≥80 & <140 | ≥70 & <80 or  ≥140 & <155 | ≥60 & <70 or  ≥155 & <170 | <60 or ≥170 | ≥80 & <140 | ≥70 & <80 or  ≥140 & <155 | ≥60 & <70 or  ≥155 & <170 | <60 or ≥170 |
| RR (rpm) | ≥15 & <25 | ≥12 & <15 or  ≥25 & <30 | ≥30 & <40 | <12 or ≥40 | ≥15 & <25 | ≥12 & <15 or  ≥25 & <30 | ≥30 & <40 | <12 or ≥40 |
| Body Temp. (º) | ≥35,7 & <37 | ≥37,5 & <38,5 or  < 35,7 | ≥38,5 | - | ≥35,7 & <37 | ≥37,5 & <38,5 or  < 35,7 | ≥38,5 | - |
| SpO_2_ (%) | ≥94 & <100 | ≥90 & <94 | ≥85 & <90 | <85 | ≥80 & <90 | ≥75 & <80 or  ≥90 & <100 | ≥70 & <75 | <70 |
| SBP (mmHg) | ≥80 & <100 | ≥70 & <80 or  ≥100 & <110 | ≥60 & <70 or  ≥110 & <120 | <60 or ≥120 | ≥80 & <100 | ≥70 & <80 or  ≥100 & <110 | ≥60 & <70 or  ≥110 & <120 | <60 or ≥120 |
| DBP (mmHg) | ≥50 & <70 | ≥40 & <50 or  ≥70 & <80 | ≥30 & <40 or  ≥80 & <90 | <30 or ≥90 | ≥50 & <70 | ≥40 & <50 or  ≥70 & <80 | ≥30 & <40 or  ≥80 & <90 | <30 or ≥90 |
| *Age group: 6 – 10 years* | | | | | | | | |
| HR (bpm) | ≥70 & <120 | ≥60 & <70 or  ≥120 & <135 | ≥50 & <60 or  ≥135 & <155 | <50 or ≥155 | ≥70 & <120 | ≥60 & <70 or  ≥120 & <135 | ≥50 & <60 or  ≥135 & <155 | <50 or ≥155 |
| RR (rpm) | ≥15 & <20 | ≥12 & <15 or  ≥20 & <30 | ≥30 & <40 | <12 or ≥40 | ≥15 & <20 | ≥12 & <15 or  ≥20 & <30 | ≥30 & <40 | <12 or ≥40 |
| Body Temp. (º) | ≥35,7 & <37 | ≥37,5 & <38,5 or  < 35,7 | ≥38,5 | - | ≥35,7 & <37 | ≥37,5 & <38,5 or  < 35,7 | ≥38,5 | - |
| SpO_2_ (%) | ≥94 & <100 | ≥90 & <94 | ≥85 & <90 | <85 | ≥80 & <90 | ≥75 & <80 or  ≥90 & <100 | ≥70 & <75 | <70 |
| SBP (mmHg) | ≥90 & <115 | ≥80 & <90 or  ≥115 & <125 | ≥70 & <80 or  ≥125 & <135 | <70 or ≥135 | ≥90 & <115 | ≥80 & <90 or  ≥115 & <125 | ≥70 & <80 or  ≥125 & <135 | <70 or ≥135 |
| DBP (mmHg) | ≥55 & <80 | ≥45 & <55 or  ≥80 & <90 | ≥35 & <45 or  ≥90 & <100 | <35 or ≥100 | ≥55 & <80 | ≥45 & <55 or  ≥80 & <90 | ≥35 & <45 or  ≥90 & <100 | <35 or ≥100 |
| *Age group: 10 – 14 years* | | | | | | | | |
| HR (bpm) | ≥60 & <100 | ≥50 & <60 or  ≥100 & <120 | ≥40 & <50 or  ≥120 & <145 | <40 or ≥145 | ≥60 & <100 | ≥50 & <60 or  ≥100 & <120 | ≥40 & <50 or  ≥120 & <145 | <40 or ≥145 |
| RR (rpm) | ≥12 & <15 | ≥10 & <12 or  ≥15 & <25 | ≥7 & <10 or  ≥25 & <35 | <7 or ≥35 | ≥12 & <15 | ≥10 & <12 or  ≥15 & <25 | ≥7 & <10 or  ≥25 & <35 | <7 or ≥35 |
| Body Temp. (º) | ≥35,7 & <37 | ≥37,5 & <38,5 or  < 35,7 | ≥38,5 | - | ≥35,7 & <37 | ≥37,5 & <38,5 or  < 35,7 | ≥38,5 | - |
| SpO_2_ (%) | ≥94 & <100 | ≥90 & <94 | ≥85 & <90 | <85 | ≥80 & <90 | ≥75 & <80 or  ≥90 & <100 | ≥70 & <75 | <70 |
| SBP (mmHg) | ≥100 & <125 | ≥90 & <100 or  ≥125 & <135 | ≥80 & <90 or  ≥135 & <150 | <80 or ≥150 | ≥100 & <125 | ≥90 & <100 or  ≥125 & <135 | ≥80 & <90 or  ≥135 & <150 | <80 or ≥150 |
| DBP (mmHg) | ≥60 & <85 | ≥50 & <60 or  ≥85 & <95 | ≥40 & <50 or  ≥95 & <105 | <40 or ≥105 | ≥60 & <85 | ≥50 & <60 or  ≥85 & <95 | ≥40 & <50 or  ≥95 & <105 | <40 or ≥105 |
| *Age group: 14 – 18 years* | | | | | | | | |
| HR (bpm) | ≥50 & <100 | ≥40 & <50 or  ≥100 & <120 | ≥35 & <40 or  ≥120 & <145 | <35 or ≥145 | ≥50 & <100 | ≥40 & <50 or  ≥100 & <120 | ≥35 & <40 or  ≥120 & <145 | <35 or ≥145 |
| RR (rpm) | ≥12 & <15 | ≥10 & <12 or  ≥15 & <25 | ≥7 & <10 or  ≥25 & <35 | <7 or ≥35 | ≥12 & <15 | ≥10 & <12 or  ≥15 & <25 | ≥7 & <10 or  ≥25 & <35 | <7 or ≥35 |
| Body Temp. (º) | ≥35,7 & <37 | ≥37,5 & <38,5 or  < 35,7 | ≥38,5 | - | ≥35,7 & <37 | ≥37,5 & <38,5 or  < 35,7 | ≥38,5 | - |
| SpO_2_ (%) | ≥94 & <100 | ≥90 & <94 | ≥85 & <90 | <85 | ≥94 & <100 | ≥90 & <94 | ≥85 & <90 | <85 |
| SBP (mmHg) | ≥100 & <130 | ≥90 & <100 or  ≥130 & <145 | ≥80 & <90 or  ≥145 & <160 | <80 or ≥160 | ≥100 & <130 | ≥90 & <100 or  ≥130 & <145 | ≥80 & <90 or  ≥145 & <160 | <80 or ≥160 |
| DBP (mmHg) | ≥60 & <85 | ≥50 & <60 or  ≥85 & <95 | ≥40 & <50 or  ≥95 & <105 | <40 or ≥105 | ≥60 & <85 | ≥50 & <60 or  ≥85 & <95 | ≥40 & <50 or  ≥95 & <105 | <40 or ≥105 |

HR: heart rate; RR: respiration rate; SpO2: oxygen saturation; Temp: temperature; SBP: Systolic Blood Pressure; DBP: Diastolic Blood Pressure. **Cyanotic congenital heart disease (CHD)** include the following defects: common arterial trunk (before corrective surgery), double outlet right ventricle (before corrective surgery), double outlet left ventricle, transposition of the great arteries, double inlet ventricle, pulmonary valve atresia, pulmonary valve stenosis, tricuspid valve stenosis, Ebstein’s anomaly, hypoplastic right heart syndrome, hypoplastic left heart syndrome, pulmonary infundibular stenosis, subaortic stenosis, atresia of aorta, interruption of the aortic arch, atresia of pulmonary artery, stenosis of pulmonary artery, congenital pulmonary arteriovenous malformation and total anomalous pulmonary venous connection.

**REFERENCES**

1. Park SJ, Cho K-J, Kwon O, Park H, Lee Y, Shim WH, Park CR, Jhang WK. Development and validation of a deep-learning-based pediatric early warning system: a single-center study. *Biomedical Journal* (2021) doi: 10.1016/j.bj.2021.01.003

2. Comoretto RI, Azzolina D, Amigoni A, Stoppa G, Todino F, Wolfler A, Gregori D, Group OBOTTipnS. Predicting Hemodynamic Failure Development in PICU Using Machine Learning Techniques. *Diagnostics* (2021) **11**:1299. doi: 10.3390/diagnostics11071299

3. Ruiz VM, Goldsmith MP, Shi L, Simpao AF, Gálvez JA, Naim MY, Nadkarni V, Gaynor JW, Tsui F (Rich). Early prediction of clinical deterioration using data-driven machine learning modeling of electronic health records. *The Journal of Thoracic and Cardiovascular Surgery* (2021) doi: 10.1016/j.jtcvs.2021.10.060

4. Gu Y, Li Q, Lin R, Jiang W, Wang X, Zhou G, Su J, Fan X, Gao P, Jin M, et al. Prognostic Model to Predict Postoperative Adverse Events in Pediatric Patients With Aortic Coarctation. *Frontiers in Cardiovascular Medicine* (2021) **8**:672627. doi: 10.3389/fcvm.2021.672627

5. Zeng X, Hu Y, Shu L, Li J, Duan H, Shu Q, Li H. Explainable machine-learning predictions for complications after pediatric congenital heart surgery. *Scientific Reports* (2021) **11**:17244. doi: 10.1038/s41598-021-96721-w

6. Mayampurath A, Jani P, Dai Y, Gibbons R, Edelson D, Churpek MM. A Vital Sign-Based Model to Predict Clinical Deterioration in Hospitalized Children*. *Pediatric Critical Care Medicine* (2020) **21**:820–826. doi: 10.1097/pcc.0000000000002414

7. Izquierdo L, Niño LF, Rojas JS. Modeling the vital sign space to detect the deterioration of patients in a pediatric intensive care unit. *16th International Symposium on Medical Information Processing and Analysis* (2020) **11583**:115830Q–115830Q–9. doi: 10.1117/12.2579629

8. Gorham TJ, Rust S, Rust L, Kuehn S, Yang J, Lin JS, Hoffman J, Huang Y, Lin S, McClead R, et al. The Vitals Risk Index—Retrospective Performance Analysis of an Automated and Objective Pediatric Early Warning System. *Pediatric Quality & Safety* (2020) **5**:e271. doi: 10.1097/pq9.0000000000000271

9. Zeng X, An J, Lin R, Dong C, Zheng A, Li J, Duan H, Shu Q, Li H. Prediction of complications after paediatric cardiac surgery. *European Journal of Cardio-Thoracic Surgery* (2019) **57**:350–358. doi: 10.1093/ejcts/ezz198

10. Ruiz VM, Saenz L, Lopez-Magallon A, Shields A, Ogoe HA, Suresh S, Munoz R, Tsui FR. Early prediction of critical events for infants with single-ventricle physiology in critical care using routinely collected data. *The Journal of Thoracic and Cardiovascular Surgery* (2019) **158**:234–243.e3. doi: 10.1016/j.jtcvs.2019.01.130

11. Berry JG, Johnson C, Crofton C, Staffa SJ, DiTillio M, Leahy I, Salem J, Rangel SJ, Singer SJ, Ferrari L. Predicting Postoperative Physiologic Decline After Surgery. *Pediatrics* (2019) **143**:e20182042. doi: 10.1542/peds.2018-2042

12. Kwon JM, Jeon KH, Lee M, Kim KH, Park J, Oh BH. Deep learning algorithm to predict need for critical care in pediatric emergency departments. *Pediatric Emergency Care* (2021) **37**:E988–E994. doi: 10.1097/PEC.0000000000001858

13. Rubin J, Potes C, Xu-Wilson M, Dong J, Rahman A, Nguyen H, Moromisato D. An ensemble boosting model for predicting transfer to the pediatric intensive care unit. *International Journal of Medical Informatics* (2018) **112**:15–20. doi: 10.1016/j.ijmedinf.2018.01.001

14. Matam BR, Duncan H, Lowe D. Machine learning based framework to predict cardiac arrests in a paediatric intensive care unit. *Journal of Clinical Monitoring and Computing* (2019) **33**:713–724. doi: 10.1007/s10877-018-0198-0

15. Wellner B, Grand J, Canzone E, Coarr M, Brady PW, Simmons J, Kirkendall E, Dean N, Kleinman M, Sylvester P. Predicting Unplanned Transfers to the Intensive Care Unit: A Machine Learning Approach Leveraging Diverse Clinical Elements. *JMIR Medical Informatics* (2017) **5**:e45. doi: 10.2196/medinform.8680

16. Potes C, Conroy B, Xu-Wilson M, Newth C, Inwald D, Frassica J. A clinical prediction model to identify patients at high risk of hemodynamic instability in the pediatric intensive care unit. *Critical Care* (2017) **21**:282. doi: 10.1186/s13054-017-1874-z

17. Rusin CG, Acosta SI, Shekerdemian LS, Vu EL, Bavare AC, Myers RB, Patterson LW, Brady KM, Penny DJ. Prediction of imminent, severe deterioration of children with parallel circulations using real-time processing of physiologic data. *The Journal of Thoracic and Cardiovascular Surgery* (2016) **152**:171–177. doi: 10.1016/j.jtcvs.2016.03.083

18. Zhai H, Brady P, Li Q, Lingren T, Ni Y, Wheeler DS, Solti I. Developing and evaluating a machine learning based algorithm to predict the need of pediatric intensive care unit transfer for newly hospitalized children. *Resuscitation* (2014) **85**:1065–1071. doi: 10.1016/j.resuscitation.2014.04.009

19. Lee B, Kim K, Hwang H, Kim YS, Chung EH, Yoon J-S, Cho HJ, Park JD. Development of a machine learning model for predicting pediatric mortality in the early stages of intensive care unit admission. *Scientific Reports* (2021) **11**:1263. doi: 10.1038/s41598-020-80474-z

20. Aczon MD, Ledbetter DR, Laksana E, Ho L v, Wetzel RC. Continuous Prediction of Mortality in the PICU: A Recurrent Neural Network Model in a Single-Center Dataset*. *Pediatric Critical Care Medicine* (2021) **22**:519–529. doi: 10.1097/pcc.0000000000002682

21. Hong S, Hou X, Jing J, Ge W, Zhang L. Predicting Risk of Mortality in Pediatric ICU Based on Ensemble Step-Wise Feature Selection. *Health Data Science* (2021) **2021**:1–7. doi: 10.34133/2021/9365125

22. Prince RD, Akhondi-Asl A, Mehta NM, Geva A. A Machine Learning Classifier Improves Mortality Prediction Compared With Pediatric Logistic Organ Dysfunction-2 Score: Model Development and Validation. *Critical Care Explorations* (2021) **3**:e0426. doi: 10.1097/cce.0000000000000426

23. Bertsimas D, Zhuo D, Dunn J, Levine J, Zuccarelli E, Smyrnakis N, Tobota Z, Maruszewski B, Fragata J, Sarris GE. Adverse Outcomes Prediction for Congenital Heart Surgery: A Machine Learning Approach. *World Journal for Pediatric and Congenital Heart Surgery* (2021) **12**:453–460. doi: 10.1177/21501351211007106

24. Hu Y, Gong X, Shu L, Zeng X, Duan H, Luo Q, Zhang B, Ji Y, Wang X, Shu Q, et al. Understanding risk factors for postoperative mortality in neonates based on explainable machine learning technology. *Journal of Pediatric Surgery* (2021) **56**:2165–2171. doi: 10.1016/j.jpedsurg.2021.03.057

25. Jalali A, Lonsdale H, Do N, Peck J, Gupta M, Kutty S, Ghazarian SR, Jacobs JP, Rehman M, Ahumada LM. Deep Learning for Improved Risk Prediction in Surgical Outcomes. *Scientific Reports* (2020) **10**:9289. doi: 10.1038/s41598-020-62971-3

26. Kim SY, Kim S, Cho J, Kim YS, Sol IS, Sung Y, Cho I, Park M, Jang H, Kim YH, et al. A deep learning model for real-time mortality prediction in critically ill children. *Critical Care* (2019) **23**:279. doi: 10.1186/s13054-019-2561-z

27. Miller R, Tumin D, Cooper J, Hayes D, Tobias JD. Prediction of mortality following pediatric heart transplant using machine learning algorithms. *Pediatric Transplantation* (2019) **23**:e13360. doi: 10.1111/petr.13360

28. Williams JB, Ghosh D, Wetzel RC. Applying Machine Learning to Pediatric Critical Care Data. *Pediatric Critical Care Medicine* (2018) **19**:599–608. doi: 10.1097/pcc.0000000000001567

29. Ho L v, Ledbetter D, Aczon M, Wetzel R. The Dependence of Machine Learning on Electronic Medical Record Quality. *AMIA Annu Symp Proc* (2017) **2017**:883–891.

30. Aczon M, Ledbetter D, Ho L, Gunny A, Flynn A, Williams J, Wetzel R. Dynamic Mortality Risk Predictions in Pediatric Critical Care Using Recurrent Neural Networks. *arXiv* (2017)
